# ﻿Taxonomic review of the Oriental genus *Phylladothrips* Priesner (Thysanoptera, Phlaeothripidae)

**DOI:** 10.3897/zookeys.1185.113895

**Published:** 2023-11-29

**Authors:** Lihong Dang, Yiyan An, Shuji Okajima, Laurence A. Mound

**Affiliations:** 1 School of Bioscience and Engineering, Shaanxi University of Technology, Hanzhong, 723000, China Shaanxi University of Technology Hanzhong China; 2 Shaanxi Province Key Laboratory of Bioresources, Hanzhong, 723000, China Shaanxi Province Key Laboratory of Bioresources Hanzhong China; 3 Qinba Mountain Area Collaborative Innovation Center of Bioresources Comprehensive Development, Hanzhong, 723000, China Qinba Mountain Area Collaborative Innovation Center of Bioresources Comprehensive Development Hanzhong China; 4 Qinba State Key Laboratory of Biological Resources and Ecological Environment (Incubation), Hanzhong, 723000, China Qinba State Key Laboratory of Biological Resources and Ecological Environment (Incubation) Hanzhong China; 5 Tokyo University of Agriculture, 1737 Funako, Atsugi, Kanagawa, 243-0034, Japan Tokyo University of Agriculture Kanagawa Japan; 6 Australian National Insect Collection CSIRO, PO Box 1700, Canberra, ACT 2601, Australia Australian National Insect Collection CSIRO Canberra Australia

**Keywords:** Fungus-feeding, identification key, *Phylladothripstrisetae*, *P.selangor*, taxonomy, thrips

## Abstract

Species of the Oriental subtropical and tropical genus *Phylladothrips* of fungus-feeding thrips exhibit some diagnostic character states, usually with abdominal tergite VIII bearing two pairs of wing-retaining setae and male tergite IX setae S2 about as long as S1. These species are quite small, and the maxillary stylets unusually broad for Phlaeothripinae. *Phylladothripstrisetae***sp. nov.** from Xizang, China and *P.selangor***sp. nov.** from Selangor, Malaysia are described, and *P.fasciae* is newly recorded from China. All 11 species in this genus are revised with an illustrated key.

## ﻿Introduction

Species of the Asian mainly tropical genus *Phylladothrips* are small-sized fungus-feeding thrips that live on dead leaves and branches, or at the base of grasses. They share this habitat with several other fungus-feeding Phlaeothripinae, including species of *Apelaunothrips* and *Stigmothrips* (Okajima, 1988), as well as some in the genera *Adraneothrips* and *Holothrips*. The original author of the genus, Priesner (1933), considered that it was related to plant feeding thrips in the tribe Haplothripini, but all known species of *Phylladothrips* have abdominal tergite VIII with one or two pairs of wing-retaining setae. This is a particularly unusual character state that is known only in the fungus-feeding species of three Phlaeothripinae genera *Lizalothrips*, *Propesolomonthrips* and *Solomonthrips*. These genera are recorded widely from northern Australia, Solomon Islands, the Philippines, Indonesia and Fiji. However, all members of these genera have the typical slender maxillary stylets (scarcely 3 μm wide) found in Phlaeothripinae. In contrast, the species of *Phylladothrips* have broader maxillary stylets (almost 5 μm wide), a character state shared only with species of *Docessissophothrips* genus-group and *Apelaunothrips* amongst the Phlaeothripinae. These stylets are only slightly more slender than the stylets of some Idolothripinae species, that is, they are slightly less than 5 μm wide whereas in Idolothripinae they are always more than 5 μm. A further character state complication is that in males of sub-family Phlaeothripinae the tergite IX setae S2 are usually shorter and stouter than setae S1, whereas in males of Idolothripinae setae S1 and S2 are similar in size, such as *Ophthalmothrips* (Li et al. 2022). Males of *Phylladothrips* and *Solomonthrips* share the condition of S2 setae with Idolothripinae, although the only known male of *Propesolomonthrips* has the Phlaeothripinae condition. These genera emphasize the problems involved in the sub-family classification of Phlaeothripidae and suggest that Phlaeothripinae and Idolothripinae are not sister-groups. An alternative interpretation of the confused pattern of character states might be that *Phylladothrips* and *Holothrips* represent a reversal from hyphal feeding Phlaeothripinae ancestors to feeding on spores, possibly small, thin-walled conidia that breakdown when specimens are prepared for slide mounting.

Nine species are currently included in the genus *Phylladothrips* (ThripsWiki 2023), and in describing seven of these Okajima (1988) presented a generic diagnosis and key to all nine species. The only other published mentions of the genus are by Ananthakrishnan (1971), Reyes (1994) and Dang et al. (2014), but none of these involved extensive comments. Of the nine species, *P.fasciae* Okajima, *P.gracilis* Okajima and *P.lateralis* Okajima were described from Indonesia, *P.pallidus* Okajima known only from Taiwan, *P.bispinosus* (Okajima) only from the Philippines, and *P.niger* Okajima only from Malaysia. The type species, *P.karnyi* Priesner, was described from Java, Indonesia, and subsequently recorded from southern India (Ananthakrishnan 1971). Two species are recorded more widely, *P.pictus* Okajima from Japan, Taiwan and the Philippines, and *P.similis* Okajima from Thailand, Malaysia, Indonesia and the Philippines. Here we newly record *P.fasciae* from Xizang, China, and describe *Phylladothripstrisetae* sp. nov. from Xizang, and *P.selangor* sp. nov. from Malaysia, together with an illustrated key to all 11 known species in the genus.

## ﻿Material and methods

The descriptions, photomicrograph images and drawings were produced from slide-mounted specimens with Nikon Eclipse 80i microscopes. Images were prepared with a Leica DM2500 using DIC illumination, and processed with Automontage and Adobe Photoshop v.7.0. The abbreviations used for the pronotal setae are as follows: **am** – anteromarginal, aa – anteroangular, ml – midlateral, epim – epimeral, pa – posteroangular. The unit of measurement in this study is the micrometre (μm). Most specimens studied here are available in the School of Bioscience and Engineering, Shaanxi University of Technology (**SNUT**), Hanzhong, China, the Australian National Insect Collection (**ANIC**), CSIRO, Canberra, Australia, and Tokyo University of Agriculture (**TUA**), Tokyo, Japan. Further slides were studied on loan from the Senckenberg Museum, Frankfurt (**SMF**).

## ﻿Taxonomy

### ﻿ Phylladothrips

Taxon classificationAnimaliaThysanopteraPhlaeothripidae

Priesner

D9914420-380C-5757-BACF-396019F80026

Phylladothrips Priesner, 1933: 79. Type species Phylladothripskarnyi Priesner, 1933, by monotypy.
Paradexiothrips Okajima, 1984: 730. Type species Paradexiothripsbispinosus Okajima, 1984, by monotypy. Synonymised by Okajima 1988: 707.

#### Note.

**Figure 1.**
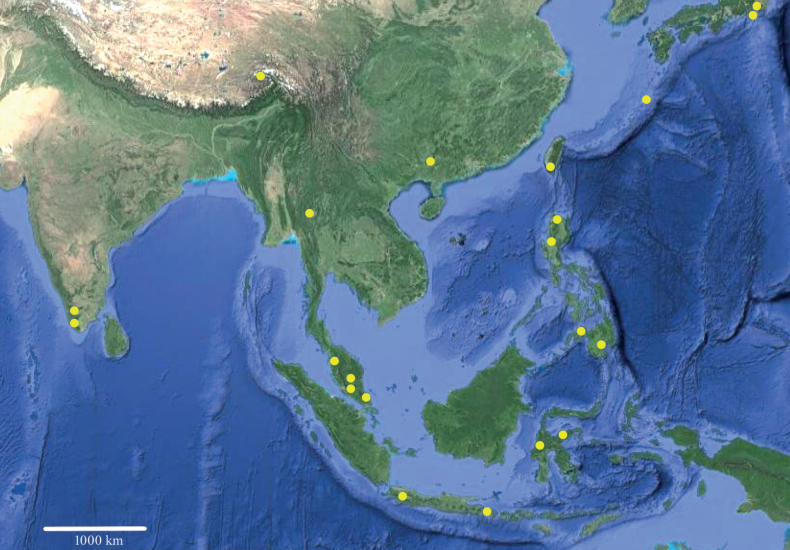
Distribution sites of *Phylladothrips* species – indicated by yellow circles.

Most species of *Phylladothrips* are found in the tropic and subtropic regions of Asia (Fig. 1). However, although described from Taiwan, the distribution of *P.pictus* extends to the temperate region, where this species was recorded from the Ryukyu Islands, the Izu Islands and Honshu of Japan (Okajima 2006). Three species of *Phylladothrips* are found in the subtropics of southern China – Guangxi and southeast of Xizang, where the fauna shares many thrips taxa with Southeast Asian countries and Japan (Dang et al. 2014). The area between mainland China and Australia is species-rich for thrips, not only both fungal feeding and plant feeding species. At present it is impossible to detect any distribution patterns due to limited exploration.

#### Diagnosis.

**Figures 2–5.**
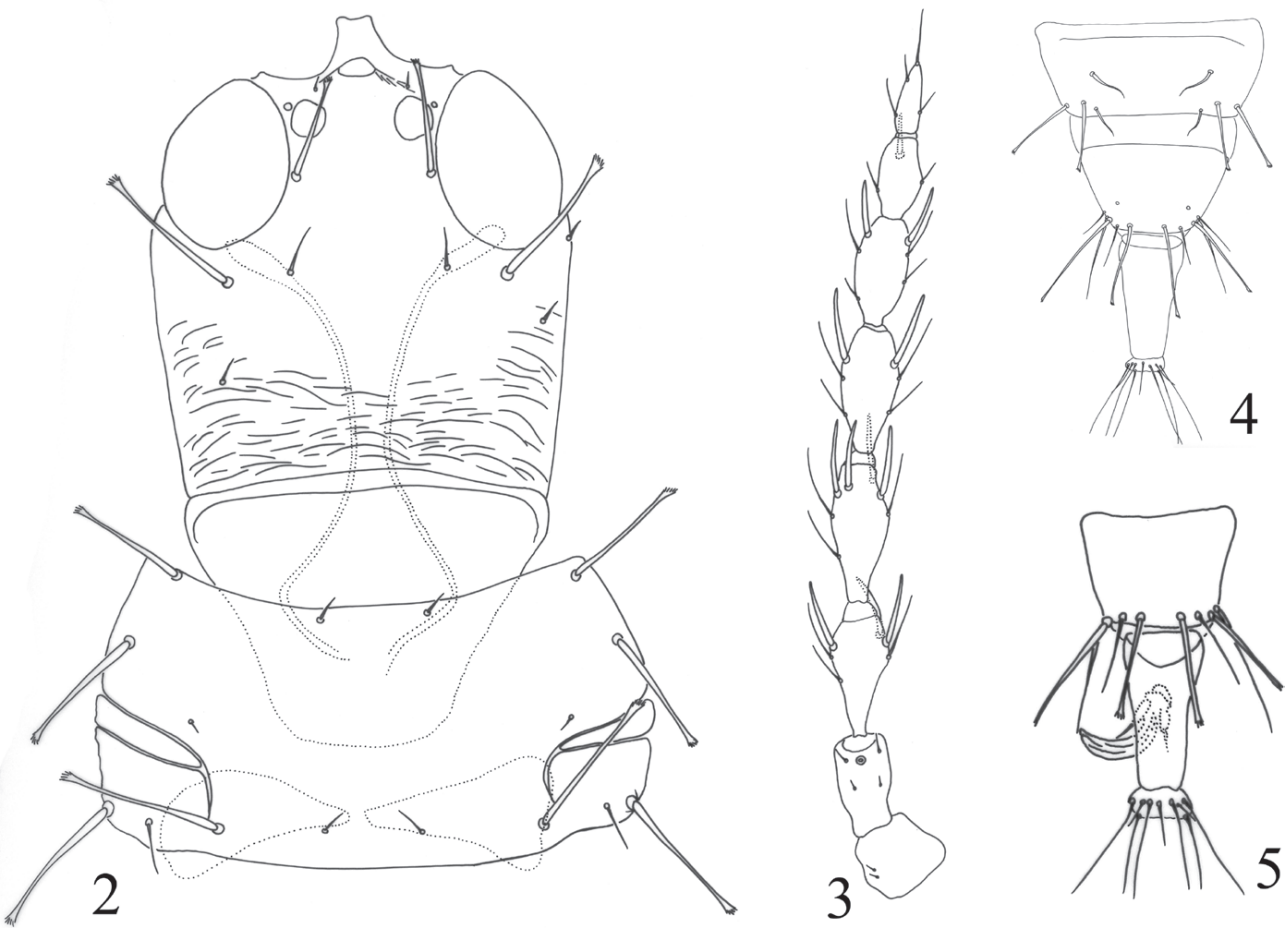
*Phylladothrips* species. *P.pallidus* (**2–4**) **2** head and pronotum **3** antenna **4** tergites VIII–X, female; *P.pictus* (**5**) **5** tergites IX–X, male.

**Figures 6–15.**
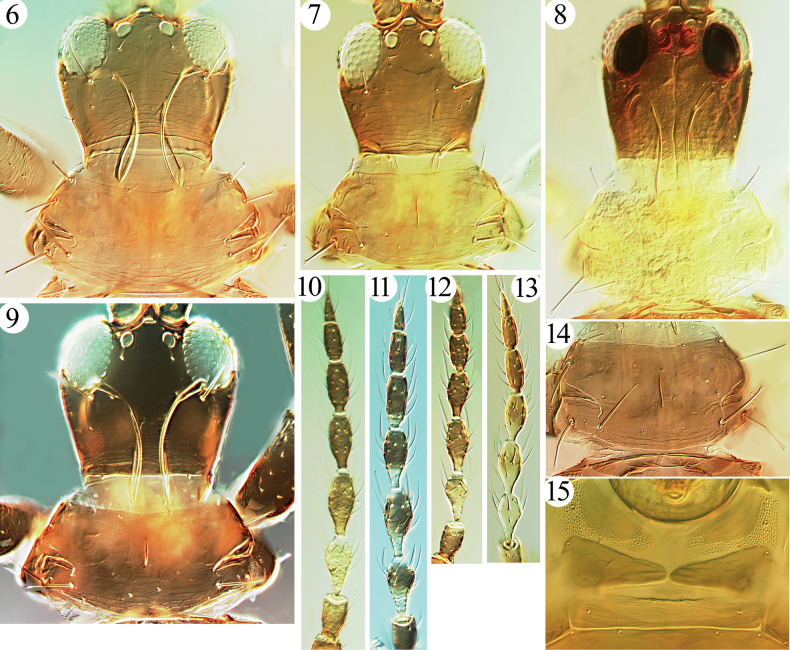
*Phylladothrips* species. Head and pronotum (**6–9**) **6***P.niger***7***P.selangor* sp. nov. **8***P.pictus***9***P.trisetae* sp. nov.; antennae (**10–13**) **10***P.pictus***11***P.trisetae* sp. nov. **12***P.fasciae***13***P.selangor* sp. nov.; pronotum (**14**) **14***P.fasciae*; basantra and mesopresternum (**15**) **15***P.selangor* sp. nov.

**Figures 16–23.**
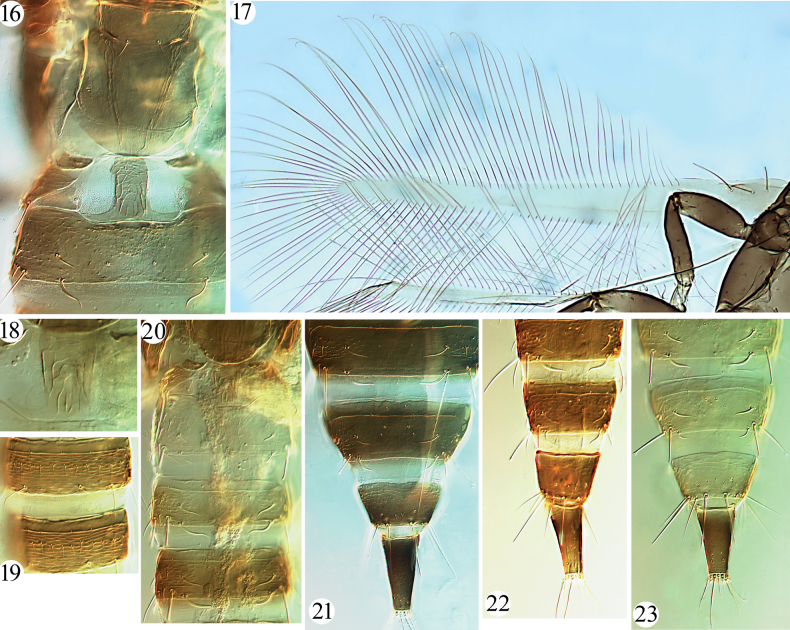
*Phylladothrips* species. *P.trisetae* sp. nov. (**16, 17**) **16** metanotum, pelta and tergite II **17** fore wing; *P.fasciae* (**18–20**) **18** pelta **19** sternites V–VI **20** pelta and tergites II–IV; tergites VII–X (**21–23**) **21***P.trisetae* sp. nov. **22***P.fasciae***23***P.selangor* sp. nov.

Small-sized fungus-feeders. Head a little wider than long, usually distinctly constricted at base (Figs 2–9); eyes large, longer than half length of head; postocellar setae usually slender, sometimes long and expanded at apex (Fig. 2); postocular setae well developed, expanded at apex; mouth-cone short and rounded, maxillary stylets broad, retracted far into head capsule, maxillary bridge weakly present or absent; antennae 8-segmented, II with campaniform sensorium on apical half of segment, III and IV with 1+2 and 2+2 sense cones respectively (Fig. 3). Pronotal am setae reduced, sometimes aa weak as well; major setae expanded at apex (Figs 2–9, 14); notopleural sutures incomplete; basantra scarcely present, or absent (Fig. 15); meso- and metanotum with weak setae and sculpture, metanotum median setal pair usually relatively long but pointed at apex; mesopresternum eroded medially (Fig. 15); metathoracic sternopleural sutures absent; fore tarsi unarmed; fore wings weakly constricted medially, without duplicated cilia (Fig. 17). Pelta hat-shaped (Figs 16, 18, 20), without campaniform sensilla; tergites II–VIII with two pairs of wing-retaining setae (Figs 20–23), sometimes VIII with only one posterior pair developed; accessory setae on tergite IX usually elongate but slender, S2 on male tergite IX well developed, about as long as S1 or a little longer (Fig. 5); tube shorter than head, anal setae usually shorter than tube; male sternite without pore plate.

### ﻿Key to *Phylladothrips* species worldwide

**Table:** 

1	Postocellar setae expanded at apex and elongate (Fig. 2)	**2**
–	Postocellar setae pointed at apex, short or long (Figs 6–9)	**3**
2	Pronotum with three pairs of major setae expanded at apex, am and aa reduced; abdominal tergite VIII with one posterior pair of wing-retaining setae; antennal segments brown with III pale at extreme base	***P.bispinosus***
–	Pronotum with four pairs of major setae expanded at apex, am reduced and pointed at apex (Fig. 2); abdominal tergite VIII with two pairs of wing-retaining setae; antennal segment III uniformly yellow, IV–V brown with extreme base pale, VI–VIII brown	***P.pallidus***
3	Head largely yellow	**4**
–	Head uniformly brown (Figs 6–9)	**5**
4	Tube bicolored, brown but yellow on anterior half; head yellow with anterior margin brown	***P.gracilis***
–	Tube largely brown with extreme base pale; head yellow with anterior and lateral margins brown	***P.lateralis***
5	Body uniformly brown	**6**
–	Body bicolored yellow and brown, at least abdominal segment II yellow	**8**
6	Pronotal am and aa setae minute, much shorter than ml (Fig. 9)	***P.trisetae* sp. nov.**
–	Pronotal am minute and pointed at apex, but aa long and expanded at apex	**7**
7	Cheeks almost parallel	P.karnyi ^*^
–	Cheeks distinctly narrowed towards base (Fig. 6)	***P.niger***
8	Body largely brown, only abdominal segment II yellow	***P.fasciae***
–	At least abdominal segments II–III yellow	**9**
9.	Abdominal segment II–III yellow, IV–V and IX brown, VI–VIII brown to brownish yellow; mid femora brown, concolorous with pterothorax	***P.similis***
–	Abdominal segments II–IX yellow (Fig. 23), sometimes IV–IX brownish yellow, or tergites III–VII each with a brown marking anteriorly; mid femora yellow, or extreme base brownish yellow	**10**
10	Prothorax yellow (Fig. 8); antennae brown with only segment III yellow (Fig. 10)	***P.pictus***
–	Prothorax brown, concolorous with head (Fig. 7); antennal segment III yellow, IV–V brown with base yellow, VI–VIII brown (Fig. 13)	***P.selangor* sp. nov.**

### ﻿ Phylladothrips bispinosus

Taxon classificationAnimaliaThysanopteraPhlaeothripidae

(Okajima)

334F9B2E-D834-5E4A-97B6-BD70D9A0886C

Paradexiothrips bispinosus Okajima, 1984: 731.

#### Material examined.

***Holotype***, ♀ (TUA), Japan, Mindanao, Mt. Apo, Agko, on dead Palmaceae leaves, 4.viii.1979 (S. Okajima); ***paratypes***, 1♀1♂ (TUA), with the same data as holotype.

#### Comments.

Described from Mindanao, Philippines on dead Arecaceae leaves, this species was transferred to *Phylladothrips* from *Paradexiothrips* by Okajima (1988). However, it can be distinguished by tergite VIII with only one posterior pair of wing-retaining setae. It is similar to *P.pallidus* described from Taiwan in having well-developed postocellar setae with apex expanded (Fig. 2), but their differences are given in the key. In addition, the broader stylets are somewhat similar to those of Idolothripinae species, and Okajima (1984) recorded lots of small fungal spores in the gut of the holotype female. Paratype males of this species have been studied, and setae S2 on tergite IX are short, a condition that is non-typical in this genus.

### ﻿ Phylladothrips fasciae

Taxon classificationAnimaliaThysanopteraPhlaeothripidae

Okajima

B240466F-A9A8-570E-A087-EB80BA4D0FC8

Figs 12 , 14 , 18–20 , 22

Phylladothrips fasciae Okajima, 1988: 709.

#### Material examined.

***Holotype***, ♀ (TUA), Indonesia, Central Sulawesi, on dead leaves and branches, 16.viii.1984 (S. Okajima); ***paratypes***, 1♀1♂ (TUA), with the same data as holotype; 1♀1♂ (SNUT), CHINA, Xizang, Motuo County, on dry leaves and grasses, 20.vii.2022 (Y.Q. Li).

#### Comments.

This species, described originally from Sulawesi, Indonesia on dead leaves and branches, can be distinguished easily by abdominal tergite II yellow in contrast to the rest of the brown body. By comparing the types of *P.fasciae*, one female and male from Xizang are here identified as this species, representing the first record of this species from China. The male has some different characters, such as abdominal tergite III yellowish brown (Fig. 20), between clear yellow II and brown IV, fore and hind femora clear yellow, and S2 (75 μm) slightly longer than S1 (65 μm) on tergite IX (Fig. 22).

### ﻿ Phylladothrips gracilis

Taxon classificationAnimaliaThysanopteraPhlaeothripidae

Okajima

6BCE55B5-9F2A-5BFE-B4B7-F264356F7A6E

Phylladothrips gracilis Okajima, 1988: 711.

#### Material examined.

***Holotype***, ♀ (TUA), Indonesia, South Sulawesi, Karaenta Forest, on dead Palmae, 6.viii.1984 (S. Okajima); ***paratypes***, 1♀1♂ (TUA), with the same data as holotype.

#### Comments.

Described from Sulawesi, Indonesia on dead Arecaceae branches, this species is similar in body coloration with another Indonesian species, *P.lateralis* from Bali Island. Together they can be distinguished easily from other *Phylladothrips* species by the largely yellow body, especially yellow heads. However, *P.gracilis* has unusual pale antennal segments I–II in contrast to *P.lateralis* with I–II brown.

### ﻿ Phylladothrips karnyi

Taxon classificationAnimaliaThysanopteraPhlaeothripidae

Priesner

7C263379-26FD-55FF-B137-98EECA62A4CD

Phylladothrips karnyi Priesner, 1933: 80.

#### Note.

The type species of the genus was described from a single female taken at Java, Indonesia on leaves of *Ammomum* sp. This specimen was studied by Okajima (1988), who indicated that the species can be distinguished from *P.niger* from Malaysia only by the parallel cheeks of the head although they are very similar in body coloration and shape. The type specimen was not seen during the present studies.

### ﻿ Phylladothrips lateralis

Taxon classificationAnimaliaThysanopteraPhlaeothripidae

Okajima

F185A209-AB04-5C09-911C-45B7ED7476A6

Phylladothrips lateralis Okajima, 1988: 713.

#### Material examined.

***Holotype***, ♂ (TUA), Indonesia, Bali Island, on dead Palmae fronds, 29.viii.1984 (S. Okajima); ***paratype***, 1♂ (TUA), with the same data as holotype.

#### Comments.

Known only from two males taken at Gilimanuk, Bali, Indonesia on dead Arecaceae fronds, this species is similar to *P.gracilis*, with the differences between them discussed above.

### ﻿ Phylladothrips niger

Taxon classificationAnimaliaThysanopteraPhlaeothripidae

Okajima

719471BA-EEA4-5CAE-9797-30BFB889CC7E

Fig. 6

Phylladothrips niger Okajima, 1988: 714.

#### Material examined.

***Holotype***, ♀ (TUA), Malaysia, Cameron Highland, Tanah Rata, on dead leaves, 2.iii.1976 (W. Suzuki); ***paratypes***, 1♀1♂ (TUA), with the same data as holotype; 1♀1♂ (ANIC), MALAYSIA, Genting Highlands, on dead wood and leaves, 28.ix.1973 (L. Mound); 1♀1♂ (ANIC), MALAYSIA, Gunung Belumut, Johor, on dry leaves and branches, 12.viii.2009 (Y.F. Ng & X.L. Eow).

#### Comments.

Described from Tanah Rata, West Malaysia, on dead leaves and recorded from Luzon National Park, the Philippines, this is the second species after the type species with the body uniformly brown; the third is described here as a new species from Xizang. The cheeks of head are distinctly constricted towards the base (Fig. 6), distinguishing this from the type species, *P.karnyi*.

### ﻿ Phylladothrips pallidus

Taxon classificationAnimaliaThysanopteraPhlaeothripidae

Okajima

2626B53F-288C-5503-A9B5-389591A3CB43

Figs 2–4

Phylladothrips pallidus Okajima, 1988: 716.

#### Material examined.

***Paratypes***, 1♀1♂ (SMF), China, Taiwan, Pintung Hsien, on dead leaves, 19.iii.1984 (S. Okajima).

#### Comments.

Described from Kenting National Park, Taiwan on dead leaves, this species is one of the two *Phylladothrips* species having postocellar setae elongate and expanded at apex (Fig. 2), while the other one is *P.bispinosus* from the Philippines. However, in *P.pallidus* there are two pairs of wing-retaining setae on tergite VIII (Fig. 4) but only one pair in *P.bispinosus*.

### ﻿ Phylladothrips pictus

Taxon classificationAnimaliaThysanopteraPhlaeothripidae

Okajima

10ED8581-9DE2-5C5B-90BA-0ED8947D5C59

Figs 5 , 8 , 10

Phylladothrips pictus Okajima, 1988: 717.

#### Material examined.

***Holotype***, ♀ (TUA), China, Taiwan, Pintung Hsien, Kenting National Park, on dead leaves, 19.iii.1984 (S. Okajima); ***paratypes***, 1♀1♂ (TUA and SMF), with the same data as holotype; 1♀ (TUA), Japan, Yokohama, Tomioka, on dead fronds of *Trachycarpus* sp., 22.ix.2019; 6♀2♂ (TUA), Japan, Ryukyu and Izu Islands, 26.x.1985 and 4.iv.1990 (S. Okajima); 1♀2♂ (SNUT), China, Guangxi, Chongzhou, Fusui County, on dead branches, 7.viii.2021 (X. Wang).

#### Comments.

Described from Kenting National Park, Taiwan, on dead leaves and branches and recorded from subtropical and temperate Japan (the Ryukyu Islands, Izu Islands and Honshu) on dead twigs, some type-specimens of this species were collected with *P.pallidus*. They are closely similar to each other in the bicolored body, but they can be distinguished by the shape of the postocellar setae, as indicated in the key, also the different color patterns, with head (Fig. 8) and abdominal tergite I brown in *P.pictus* but yellow in *P.pallidus*. One female and two males from Guangxi, China are exactly the same as the holotype and paratype specimens on loan from SMF and TUA, and this is the first record of this species from China. *Phylladothripspictus* is also similar to the new species from Malaysia, but the differences between them are discussed under the comments of this new species.

### ﻿ Phylladothrips selangor sp. nov.

Taxon classificationAnimaliaThysanopteraPhlaeothripidae

742DB151-D855-5151-AF11-B2F5BCB4EE5F

https://zoobank.org/9DC58638-0E6E-4BE6-87EB-62E37846E283

Figs 7 , 13 , 15 , 23

#### Material examined.

***Holotype***, ♀ (ANIC), Malaysia, University of Malaya, on dead palm, 08.v.2006, L. Mound. ***Paratypes***, 1♀2♂ (ANIC), with the same data as holotype.

#### Description.

**Holotype. *Female macroptera*.** Body bicoloured. Head, thorax and tube brown (Figs 7, 23), abdominal segment I–III clear yellow, IV–V yellow with brownish at lateral 1/3, VI–IX yellow, tube brown with extreme base pale. Antennal segments I–II light brown, III clear yellow, IV brown with yellow at basal half, V–VIII uniformly brown (Fig. 13); fore legs clear yellow except brown coxa, mid legs largely yellow, but coxa brown and basal half of femora light brown, hind legs uniformly yellow. Wings yellow with slightly shading.

***Head*.** Head inverted trapezoid, a little wider than long (Fig. 7); dorsal surface smooth with slightly transverse striae on basal third; eyes large, 0.4 times as long as head, postocular setae much shorter than eyes, expanded at apex (Fig. 7); cheeks constricted at base. Mouth-cone rounded, maxillary stylets broad and long (possibly retracted to postocular setae but stylets damaged in holotype, Fig. 7). Antennae 8-segmented, III–VI with basal stem, VIII slightly constricted at base, sensoria elongate, longer than half of this segment, III with 1+2, IV with 2+2, V–VI with 1+1 respectively, VII with one ventrally (Fig. 13).

***Thorax*.** Pronotum smooth with weak sculpture close to posterior margin, notopleural sutures incomplete (Fig. 7); setae am minute, aa, ml, epim and pa setae developed, expanded at apex (Fig. 7), basantra present but weak; mesonotum with weak sculpture in front part, all setae minute; mesopresternum eroded medially, reduced to two lateral plates (Fig. 15); metanotum weakly reticulate, three pairs of tiny setae on anterior angles, a pair of median setae slightly larger, pointed at apex. Legs without fore tarsal tooth. Fore wings slender, weakly constricted medially, without duplicated cilia; three pairs of subbasal setae well developed, expanded at apex, S2 about as long as S3.

***Abdomen*.** Pelta reticulate, tall hat-shaped; abdominal tergites II–VIII with two pairs of wing-retaining setae, posterior pair on tergite VIII slightly smaller than anterior pair (Fig. 23); S1–S2 on tergite IX expanded at apex, S1 slightly shorter than S2, S3 the longest, about as long as tube, pointed at apex, accessory setae between S1 and S2 elongate, slightly shorter than S1, pointed at apex (Fig. 23); tube about 0.6 times as long as head, anal setae shorter than tube; sternites II–VIII with a row of 8–15 slender setae.

***Measurements*** (holotype female in μm). Body length 1510. Head length 150, width just behind eyes 168, width at base 130; eye length 73, postocular setae length 28. Antenna length 345, segments I–VIII length (widest) 25(30), 35(25), 50(25), 60(25), 50(25), 48(20), 38(18) and 30(12), sensoria on segment III length 38. Fore wing length 615, subbasal setae length, S1 25, S2 35, S3 40. Pronotum length 100, width 200, length of pronotal setae, am 5, aa 28, ml 25, epim 45, pa 35. Pelta length 60, width at apex 50, width at base 100; tergite IX posteromarginal setae S1–S3, 75, 83, 88, accessorial setae length 63; tube length 90, basal width 55, apical width 25; anal setae length 70.

***Male macroptera*.** Similar to female; abdominal tergites IV–IX brownish yellow; S2 on tergite IX slightly longer than S1, accessory setae between S1 and S2 elongate, but shorter than S1, sternites II–VIII with a row of 8–11 slender setae.

***Measurements*** (paratype male in μm). Body length 1220. Head length 135, width just behind eyes 145, width at base 115; eye length 65, postocular setae length 25. Antenna length 330, segments I–VIII length (widest) 28(28), 35(25), 48(25), 53(25), 45(22), 40(20), 35(15) and 25(10), sensoria on segment III length 28. Fore wing length 560, subbasal setae length, S1 25, S2 30, S3 30. Pronotum length 90, width 175, length of pronotal setae, am 5, aa 25, ml 28, epim 45, pa 25. Pelta length 50, width at apex 30, width at base 60; tergite IX posteromarginal setae S1–S3, 60, 63, 90, accessorial setae length 57; tube length 75, basal width 45, apical width 25; anal setae length 70.

#### Etymology.

This species name is based on the collecting location.

#### Comments.

This new species is one of the typical bicolored species in this genus, and is closely related to *P.similis* and *P.pictus* as indicated in the key. However, it can be distinguished by the brown prothorax, whereas the prothorax is yellow in *P.similis* and *P.pictus*, despite the various abdomen color patterns.

### ﻿ Phylladothrips similis

Taxon classificationAnimaliaThysanopteraPhlaeothripidae

Okajima

E3F2CCFD-2038-5689-8C7C-D7491BBC0982

Phylladothrips similis Okajima, 1988: 719.

#### Material examined.

***Holotype***, ♀ (TUA), The Philippines, Mindanao, Mt. Apo, Agko, on dead leaves, 1.viii.1984 (S. Okajima); ***paratypes***, 1♀1♂ (TUA), with the same data as holotype.

#### Comments.

Described from Mindanao, the Philippines, on dead leaves, and recorded from Indonesia, Thailand and Western Malaysia, this species seems widespread around Southeast Aisa and has various body colorations. It is difficult to distinguish from *P.pictus* indicated in the original description, but they are different in the shape of the male aedeagus (Okajima 1988: figs 19–21). A paratype female of *P.pictus* on loan from SMF had abdominal segment II clear yellow, but III–IX brownish yellow and intermediate in colour between II and the brown tube. The color of abdominal segments of *P.similis* is II–III yellow, IV–V brown, VI–VIII brown to brownish yellow, IX and tube brown. However, a female from West Malaysia had the abdomen largely brown (Okajima 1988). Variation in coloration also exists in many fungus-feeding Phlaeothripinae, such as the related genera, *Apelaunothrips* and *Holothrips*. Sometimes the color helps to distinguish species, but sometimes there are gradual changes in different specimens of one species.

### ﻿ Phylladothrips trisetae sp. nov.

Taxon classificationAnimaliaThysanopteraPhlaeothripidae

A0CC7F52-828D-5568-B321-6CB1D3FFBD7E

https://zoobank.org/41B8BD90-2F2E-47BA-9C8B-5F44AC607E43

Figs 9 , 11 , 16–17 , 21

#### Material examined.

***Holotype***, ♀ (SNUT), China, Xizang, Motuo County, on leaf-litter, 20.vii.2022, Y.Q. Li; ***Paratypes***, 1♀3♂ (SNUT), with the same data as holotype; 1♀1♂ (ANIC), with the same data as holotype.

#### Description.

**Holotype. *Female macroptera*.** Body brown. All legs brown with tibiae yellow at extreme apices also all tarsi, fore and hind femora paler on apical 1/4. Antennal segments brown, with III–VI basal stems yellow (Fig. 11). Wings shaded with brown, body setae brown.

***Head*.** Head inverted trapezoid, a little wider than length (Fig. 9); dorsal surface smooth with slightly transverse striae on basal third; eyes large, 0.4 times as long as head, postocular setae well developed, but shorter than eyes, expanded at apex (Fig. 9); cheeks narrowing to base. Mouth-cone rounded, maxillary stylets long, retracted to postocular setae, about one-fourth of head width apart, bridge stout (Fig. 9). Antennae 8-segmented, III–VI with basal stem, VIII not constricted at base, III with 1+2 sensoria, IV with 2+2, V–VI with 1+1 respectively, VII with one ventrally (Fig. 11).

***Thorax*.** Pronotum smooth with weakly sculpture close to posterior margin, notopleural suture incomplete (Fig. 9); am and aa minute, ml, epim and pa setae developed, expanded at apex (Fig. 9), basantra present but weak; mesonotum with weak sculpture on front part, all setae minute, mesopresternum eroded medially, reduced to two lateral plates; metanotum smooth medially, with weak longitudinal reticulation laterally, three pairs of tiny setae on anterior angles, a pair of median setae slightly larger, pointed at apex (Fig. 16). Legs without fore tarsal tooth. Fore wings slender, weakly constricted medially, without duplicated cilia, three pairs of subbasal setae well developed, blunt at apex, S3 the longest (Fig. 17).

***Abdomen*.** Pelta weakly reticulate, tall hat-shaped (Fig. 16); abdominal tergites II–VIII with two pairs of wing-retaining setae (Figs 16, 21); S1–S3 on tergite IX shorter than tube, S1 shorter than S2, blunt at apex, S3 the shortest, pointed at apex, accessory setae between S1 and S2 elongate, but shorter than S1 (Fig. 21); tube about 0.6 times as long as head, anal setae much shorter than tube.

***Measurements*** (holotype female in μm). Body length 1975. Head length 190, width just behind eyes 200, width at base 160; eye length 80, postocular setae length 60. Antenna length 435, segments I–VIII length (widest) 35(35), 45(30), 55(30), 70(30), 60(25), 60(20), 45(20) and 30(10), sensoria on segment III length 25. Fore wing length 860, subbasal setae length, S1 50, S2 60, S3 85. Pronotum length 125, width 255, length of pronotal setae, am 10, aa 10, ml 20, epim 65, pa 60. Pelta length 95, width at apex 50, width at base 125; tergite IX posteromarginal setae S1–S3, 90, 100, 80, accessorial setae length 55; tube length 130, basal width 65, apical width 40; anal setae length 80.

***Male macroptera***. Similar to female; abdominal tergite IX setae S2 longer than S1 (unusual among Phlaeothripinae, accessory setae between S1 and S2 elongate, but shorter than S1.

***Measurements*** (paratype male in μm). Body length 1450. Head length 155, width just behind eyes 170, width at base 125; eye length 70, postocular setae length 50. Antenna length 350, segments I–VIII length (widest) 30(30), 40(20), 55(25), 65(25), 55(20), 50(15), 35(15) and 25(10), sensoria on segment III length 30. Fore wing length 660, subbasal setae length, S1 40, S2 50, S3 60. Pronotum length 95, width 200, length of pronotal setae, am 5, aa 5, ml 35, epim 55, pa 45. Pelta length 65, width at apex 40, width at base 90; tergite IX posteromarginal setae S1–S3, 75, 85, 80, accessorial setae length 40; tube length 100, basal width 50, apical width 25; anal setae length 55.

#### Etymology.

This species name is composed of two Latin words, *tri* and *setae*, based on its pronotum with three pairs of well-developed setae expanded at apex.

#### Comments.

This new species is similar to *P.karnyi* and *P.niger* in having the body uniformly brown, but it can easily be distinguished by having three pairs of well developed and expanded setae on the pronotum (Fig. 9). This condition also occurs in *P.bispinosus*, but it can be distinguished by the short and pointed postocellar setae (Fig. 9), in contrast to the long and expanded ones in *P.bispinosus*.

## Supplementary Material

XML Treatment for Phylladothrips

XML Treatment for Phylladothrips bispinosus

XML Treatment for Phylladothrips fasciae

XML Treatment for Phylladothrips gracilis

XML Treatment for Phylladothrips karnyi

XML Treatment for Phylladothrips lateralis

XML Treatment for Phylladothrips niger

XML Treatment for Phylladothrips pallidus

XML Treatment for Phylladothrips pictus

XML Treatment for Phylladothrips selangor

XML Treatment for Phylladothrips similis

XML Treatment for Phylladothrips trisetae

## Footnotes

Species not studied

## ﻿Additional information

### Author ORCIDs

Lihong Dang https://orcid.org/0000-0002-7571-8426

Yiyan An https://orcid.org/0009-0002-6134-9188

Shuji Okajima https://orcid.org/0000-0001-7249-671X

Laurence A. Mound https://orcid.org/0000-0002-6019-4762

### Data availability

All of the data that support the findings of this study are available in the main text.
